# ACE I/D Gene Polymorphism Can't Predict the Steroid Responsiveness in Asian Children with Idiopathic Nephrotic Syndrome: A Meta-Analysis

**DOI:** 10.1371/journal.pone.0019599

**Published:** 2011-05-18

**Authors:** Tian-Biao Zhou, Yuan-Han Qin, Li-Na Su, Feng-Ying Lei, Wei-Fang Huang, Yan-Jun Zhao

**Affiliations:** Department of Pediatrics, The First Affiliated Hospital of GuangXi Medical University, NanNing, China; L' Istituto di Biomedicina ed Immunologia Molecolare, Consiglio Nazionale delle Ricerche, Italy

## Abstract

**Background:**

The results from the published studies on the association between angiotensin-converting enzyme (ACE) insertion/deletion (I/D) gene polymorphism and the treatment response to steroid in Asian children with idiopathic nephrotic syndrome (INS) is still conflicting. This meta-analysis was performed to evaluate the relation between ACE I/D gene polymorphism and treatment response to steroid in Asian children and to explore whether ACE D allele or DD genotype could become a predictive marker for steroid responsiveness.

**Methodology/Principal Findings:**

Association studies were identified from the databases of PubMed, Embase, Cochrane Library and CBM-disc (China Biological Medicine Database) as of September 1, 2010, and eligible investigations were synthesized using meta-analysis method. Five investigations were identified for the analysis of association between ACE I/D gene polymorphism and steroid-resistant nephrotic syndrome (SRNS) risk in Asian children and seven studies were included to explore the relationship between ACE I/D gene polymorphism and steroid-sensitive nephrotic syndrome (SSNS) susceptibility. Five investigations were recruited to explore the difference of ACE I/D gene distribution between SRNS and SSNS. There was no a markedly association between D allele or DD genotype and SRNS susceptibility or SSNS risk, and the gene distribution differences of ACE between SRNS and SSNS were not statistically significant. II genotype might play a positive role against SRNS onset but not for SSNS (OR = 0.51, *P* = 0.02; OR = 0.95, *P* = 0.85; respectively), however, the result for the association of II genotype with SRNS risk was not stable.

**Conclusions/Significance:**

Our results indicate that D allele or DD homozygous can't become a significant genetic molecular marker to predict the treatment response to steroid in Asian children with INS.

## Introduction

Idiopathic nephrotic syndrome (INS) is the most common glomerular disease in childhood [1], and uniformly present as proteinuria, hypoalbuminemia, hyperlipidemia and gravity-dependent edema, with other features like hematuria, hypertension, and decreased glomerular filtration rate [2]. With a benign prognosis, most of INS have a satisfactory response to steroid therapy [3]. According to the clinical response to steroids, INS are divided into steroid-sensitive nephrotic syndrome (SSNS) and non-steroid-sensitive nephrotic syndrome (non-SSNS), and non-SSNS is further divided into steroid-dependent nephrotic syndrome (SDNS) and steroid-resistant nephrotic syndrome (SRNS) [4]. Age of initial presentation has an important impact on the disease distribution and the response to steroid [5]. Most of children with INS respond to corticosteroid treatment (SSNS), and about 10% of children with INS are mainly steroid-resistant (SRNS) [6]. SRNS is at risk of developing end stage renal disease [7]. In clinical practice, the best prognostic indicator for INS is whether or not the disease responds to steroid treatment [8]. Patients with SSNS or SRNS have a similar clinical manifestation, and there is no specific laboratory indicator to distinguish these two clinical entities [1]. Pathological evaluation of renal cortical tissue, by means of a renal biopsy, has traditionally been used to detect a distinction between SSNS and SRNS [1]. The pathological correlations to SSNS and SRNS are minimal change disease and focal segmental glomerulosclerosis, respectively. However, these histological diagnoses aren't always parallel to patients' clinical response to treatment. Identification of noninvasive biomarkers that accurately distinguish SSNS from SRNS would be most beneficial to the patients with SRNS, preventing their exposure to high-dose, yet ineffective steroid courses [1]. In the past years, some investigations suggested that there might be an association between angiotensin-converting enzyme (ACE) insertion/deletion (I/D) gene polymorphism and treatment response to steroid in children with INS and DD genotype or D allele might become a candidate indicator for predicting the response to corticosteroid treatment.

A variety of recent well-documented evidences indicate that the renin-angiotensin system (RAS) is involved in the pathogenesis of renal disorders [9], [10], [11], [12]. ACE, a key zinc metallopeptidase, catalyses the conversion of angiotensin I to angiotensin II, which is the main active product of the RAS [13]. An increased angiotensin II level causes deleterious effects on renal haemodynamics and induces the expression of different growth factors and cytokines, leading to tubulointerstitial fibrosis and glomerulosclerosis [14]. The ACE gene consists of either an insertion (I) allele or a deletion (D) allele forming three possible genotypes: II, ID and DD [15]. DD homozygous or D allele is associated with elevated circulating and tissue ACE activity compared to I allele [6], [16], [17], [18]. Patients with SRNS eventually receive symptomatic treatment with synergic combinations of angiotensin converting enzyme inhibitors and angiotensin II receptor blockers as they seem to produce a nonspecific decrease in the proteinuria and reduce glomerular transcapillary hydrostatic pressure as well as the synthesis of profibrotic cytokines that alter glomerular permeability [19]. There might be an association between ACE and the response to corticosteroid treatment.

The ACE I/D gene polymorphism, correlating with circulating and cellular ACE concentration [16], might be implicated in the etiology of SRNS or SSNS and has been investigated in numerous epidemiologic studies. Most of them were performed in Asian children, and only a few original investigations were conducted in Caucasians or Africans. However, the available evidence is weak for Asians at present, due to sparseness of data or disagreements among the reported investigations. The geographic and race difference is an important factor to effect the association between the gene polymorphism and the susceptibility of renal diseases. So, in this study, we only included the investigations performed in Asians.

The evidence from meta-analysis may be powerful when compared with the individual investigation. In the past years, there were some meta-analyses to explore the association of ACE I/D gene polymorphism with the susceptibility of some diseases in Asians. Some investigators [20] [21], [22] respectively took a meta-analysis to investigated the association of ACE I/D gene polymorphism with immunoglobulin A nephropathy (IgAN) risk, and found that DD homozygous was associated with an increased risk of IgAN in Asians. Ji et al [23] conducted a meta-analysis to explore the association of ACE I/D gene polymorphism with essential hypertension susceptibility in Asians and found that DD homozygous was associated with hypertension risk. Zhang et al [24] performed a meta-analysis to study the relation between ACE I/D gene polymorphism and the onset of asthma, and observed that there was an association between D allele or DD genotype and the asthma susceptibility in Asian population. Whether the ACE I/D gene polymorphism was associated with the response to corticosteroid treatment and could predict the treatment response to steroid in Asian children with INS, there was rare meta-analysis to investigate. We performed this meta-analysis to investigate whether the ACE I/D gene polymorphism could become a valuable indicator to predict the steroid responsiveness in Asian children with INS.

## Materials and Methods

### 1. Search strategy

#### 1.1 Search strategy for the association of ACE I/D gene polymorphism with SRNS risk

The relevant studies were searched from the electronic databases of PubMed, Cochrane Library and CBM-disc (China Biological Medicine Database) on September 1, 2010. (Steroid resistant nephrotic syndrome OR SRNS) AND (Angiotensin converting enzyme OR ACE) was entered into these databases mentioned above for search. Additional articles were identified through references cited in retrieved articles.

#### 1.2 Search strategy for the relationship between ACE I/D gene polymorphism and SSNS susceptibility

A systematic literature search in PubMed, Cochrane Library and CBM-disc was carried out on September 1, 2010 using (Steroid sensitive nephrotic syndrome OR SRNS) AND (Angiotensin converting enzyme OR ACE). A search of bibliographies listed in those published studies was also conducted to identify the additional publications.

#### 1.3 Search strategy for the I/D gene distribution of ACE between SRNS and SSNS

PubMed, Cochrane Library and CBM-disc were searched using (Steroid resistant nephrotic syndrome OR SRNS) AND (Steroid sensitive nephrotic syndrome OR SRNS) AND (Angiotensin converting enzyme OR ACE) as of September 1, 2010, and a search of bibliographies listed in those published studies was also performed to identify additional publications.

### 2. Inclusion and Exclusion Criteria

#### 2.1 Inclusion and Exclusion Criteria for SRNS

**Inclusion criteria:** (1) A case–control study; (2) The outcome had to be SRNS; (3) There had to be at least two comparison groups (SRNS group vs control group); (4) Investigation was conducted in children.

**Exclusion criteria:** (1) Review articles and editorials; (2) Case reports; (3) Investigation did not provide the detailed data of genotype distribution; (4) Preliminary results not on ACE I/D gene polymorphism or outcome; (5) Investigating the role ACE inhibitor to diseases; (6) Association of ACE I/D gene polymorphism with INS but not SRNS.

#### 2.2 Inclusion and Exclusion Criteria for SSNS

*Inclusion criteria:* (1) Case–control investigation; (2) The outcome must be SSNS; (3) There should have at least two comparison groups (SSNS group vs control group) in the study. (4) Study was performed in children.

*Exclusion criteria:* (1) Review articles and editorials; (2) Case reports; (3) Article did not provide the detail genotype data; (4) Investigating the association of other genes with SSNS or the relation between ACE I/D gene polymorphism and other diseases. (5) Studying the role ACE inhibitor to diseases; (6) Association of ACE I/D gene polymorphism with INS but not SSNS.

#### 2.3 Inclusion and Exclusion Criteria for studies including SRNS and SSNS

*Inclusion criteria:* (1) Case–control study; (2) The outcome included SRNS and SSNS; (3) Two comparison groups (SRNS group vs SSNS) in the report was needed. (4) Investigation was implemented in children.

*Exclusion criteria:* (1) Case reports, editorials and Review articles; (2) Investigation did not provide the detailed data of genotype distribution; (3) Results not on ACE I/D gene polymorphism or outcome; (4) Investigating the role ACE inhibitor to diseases; (5) Association of ACE I/D gene polymorphism with INS but not SRNS and SSNS.

### 3. Data extraction and synthesis

Two investigators independently extracted the following information from each eligible study: first author's surname, year of publication and the number of cases and controls for ACE genotype. Frequency of D allele was calculated for case group and control group, from the corresponding genotype distribution. The results were compared and disagreements were resolved by discussion.

### 4. Statistical Analysis

Cochrane Review Manager Version 5 (Cochrane Library, UK) was used to calculate the available data from each investigation. The pooled statistic was counted using the fixed effects model and random effects model, respectively. Results were expressed with odds ratios (OR) for dichotomous data, and 95% confidence intervals (CI) were also calculated. *P*<0.05 was required for the pooled OR to be statistically significant. I^2^ was used to test the heterogeneity among the included studies. When a *P* value<0.10 indicated a significant statistical heterogeneity across studies, the results from the random effects models would be more stable when compared with those in the fixed effects model, and the final results for our study would come from those in the random effects models. In order to avoid excessive comparisons, the OR was calculated by using three methods: method 1, allele comparison (D allele vs I allele); method 2, comparing DD homozygous with the other two combinations (DD vs DI+II); method 2, comparing II genotype with the other two combinations (II vs DD+DI). A chi-square (χ2) test using a web-based program was applied to determine if genotype distributions of the control population reported conformed to Hardy-Weinberg equilibrium (HWE; *P*<0.05 was considered significant), and the study that the genotype distributions in the controls were significantly deviated from HWE was excluded from our sensitive analysis. All descriptive data were expressed as mean ± SD.

## Results

### 1 Study characteristics

#### 1.1 Study characteristics for SRNS

Seven studies were identified for the analysis of the association between ACE I/D gene polymorphism and SRNS susceptibility. However, two investigations [6] [25] were performed in Caucasians and Africans respectively, which were excluded from our meta-analysis. Finally, five studies [4], [8], [26], [27], [28] were recruited into our investigation for the relationship between ACE I/D gene polymorphism and SRNS susceptibility (Table 1). Interestingly, all the recruited investigations were performed in children and those studies were published in English. The data of our interest were extracted: first author's surname, year of publication and the number of cases and controls for ACE genotype (Table 1). Those five investigations contained 126 case series and 604 controls. The average distribution frequency of ACE D allele in patients with SRNS was 64.40% and the average frequency in controls was 48.23%. The average distribution frequency of D allele in cases was a little increase when compared with that in control group (SRNS/control = 1.34).

**Table 1:** Characteristics of the studies evaluating the effects of ACE I/D gene polymorphism on SRNS risk.

First author, year	Case			Control			D allele (%)		P(HWE)
	DD	ID	II	DD	ID	II	Case	Control	
AI-Eisa 2001	6	2	0	25	22	1	87.50	75.00	0.124
Serdaroglu 2005	34	38	11	99	124	64	63.86	56.10	0.037
Yang 2005	0	5	6	5	22	23	22.73	32.00	0.938
Celik 2006	3	16	0	51	74	15	57.90	62.86	0.118
Tsai 2006	4	1	0	2	20	57	90.00	15.19	0.877

#### 1.2 Study characteristics for SSNS

The search yielded 11 references reporting the association of ACE I/D gene polymorphism and the onset of SSNS. One study [29] was excluded because the distribution of ACE I/D gene polymorphism was not in detail. Furthermore, one report [6] was conducted in Caucasians and two [25], [30] were in Africans, and these were excluded from our investigation for the relationship between ACE I/D gene polymorphism and SSNS risk. Seven studies [4], [8], [14], [26], [27], [28], [31] were identified for the analysis of the association between ACE I/D gene polymorphism and SSNS susceptibility in our final review (Table 2). Interestingly, all the included studies were performed in children. Five studies were published in English and two [28], [31] in Chinese. Those seven investigations contained 356 case series and 787 controls. The average distribution frequency of ACE D allele in children with SSNS was 50.97%, and the average frequency in controls was 46.68%. The average distribution frequency of D allele in cases was similar with that in control group (SSNS/control = 1.09).

**Table 2:** Characteristics of the studies evaluating the effects of ACE I/D gene polymorphism on SSNS onset.

First author, year	Case			Control			D allele (%)		P(HWE)
	DD	ID	II	DD	ID	II	Case	Control	
AI-Eisa 2000	33	8	6	25	22	1	78.72	75.00	0.124
Dang 2000	1	6	15	18	36	53	18.18	33.64	0.011
Oktem 2004	16	19	8	13	53	10	59.30	51.97	0.001
Yang 2005	5	12	12	5	22	23	37.93	32.00	0.938
Serdaroglu 2005	75	49	20	99	124	64	69.10	56.10	0.037
Celik 2006	10	39	3	51	74	15	56.73	62.86	0.118
Tsai 2006	6	2	11	2	20	57	36.84	15.19	0.877

#### 1.3 Study characteristics for studies including SRNS and SSNS

Five studies [4], [8], [26], [27], [28] were included in our investigation to explore whether the ACE I/D gene distributions in SRNS were different from those in SSNS (Table 3). Those five investigations contained 126 patients with SRNS and 291 SSNS children. The average distribution frequency of ACE D allele in patients with SRNS was 64.40%, and the average frequency in SSNS was 55.87%. The average distribution frequency of D allele in SRNS group was slightly increased when compared with that in SSNS group (SRNS/SSNS = 1.15).

**Table 3:** Gene distribution characteristics of ACE I/D gene for SRNS and SSNS.

First author, year	SRNS			SSNS			D allele (%)	
	DD	ID	II	DD	ID	II	SRNS	SSNS
AI-Eisa 2001	6	2	0	33	8	6	87.50	78.72
Serdaroglu 2005	34	38	11	75	49	20	63.86	69.10
Yang 2005	0	5	6	5	12	12	22.73	37.93
Celik 2006	3	16	0	10	39	3	57.90	56.73
Tsai 2006	4	1	0	6	2	11	90.00	36.84

### 2 Association of ACE I/D gene polymorphism with treatment response to steroid

#### 2.1 Association of ACE I/D gene polymorphism with SRNS risk

**Figure 1:**
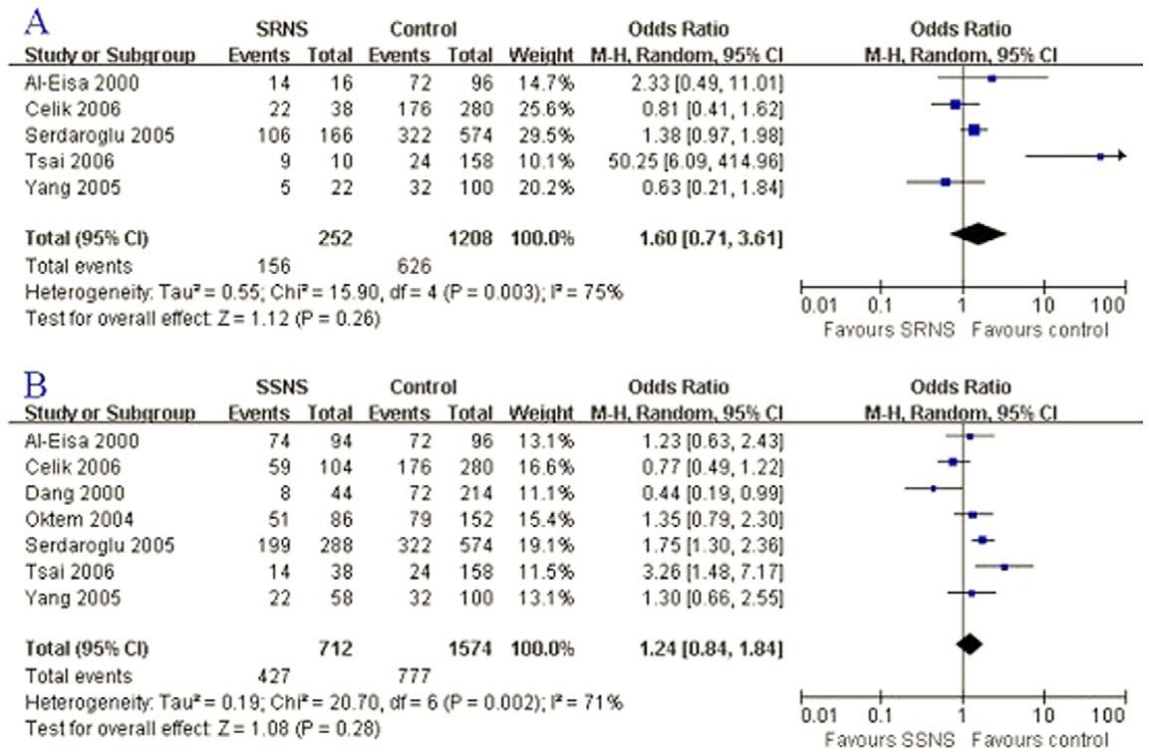
Association between D allele and SRNS susceptibility or SSNS risk. A: D allele and SRNS; B: D allele and SSNS.

**Figure 2:**
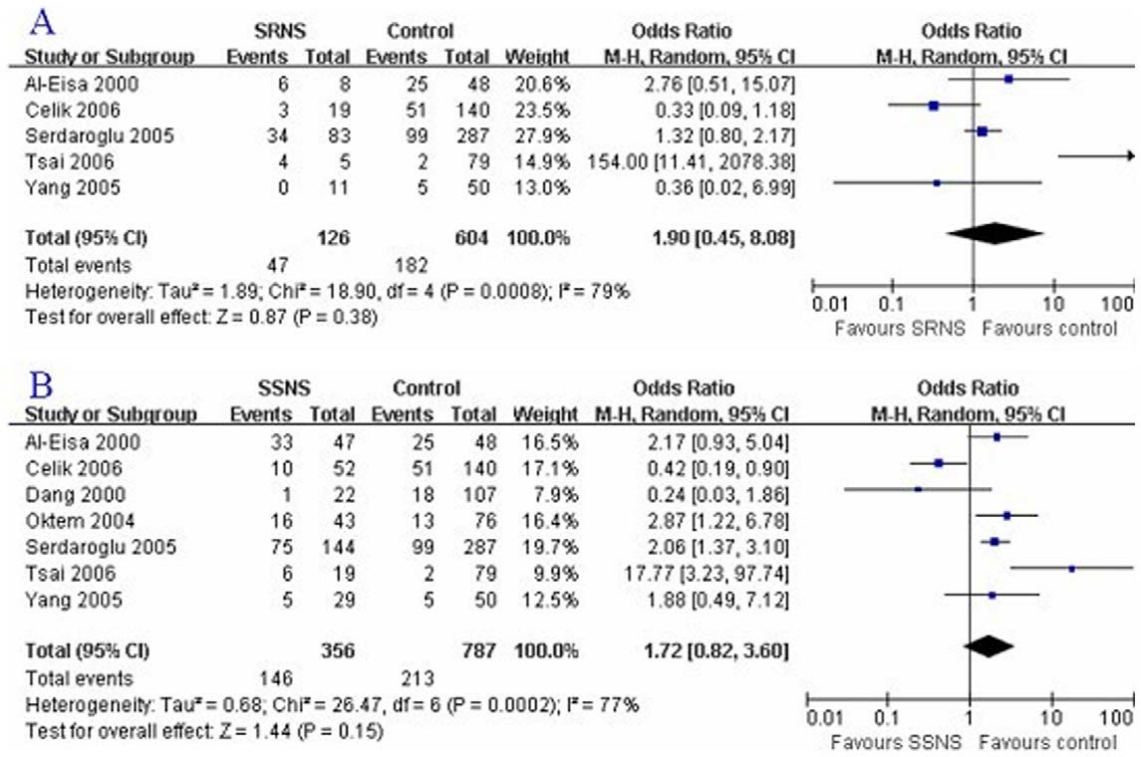
Association of DD genotype with SRNS risk and SSNS susceptibility. A: DD genotype and SRNS; B: DD genotype and SSNS.

In this meta-analysis, five investigations [4], [8], [26], [27], [28] were included into our study to explore the association of ACE I/D gene polymorphism with SRNS susceptibility in Asian children. Pooled OR were computed twice, by using the fixed effects method and the random effects method (Table 4). Random effects model were more appropriate when marked heterogeneity was present among the studies. When the fixed effects method was used to analysis those associations, the pooled OR of the ACE I/D gene polymorphism associated with SRNS risk were 1.38(95%CI: 1.04–1.83) for comparison of D allele vs I allele, 1.26(95%CI: 0.84–1.90) for comparison of combined wild homozygous and variant homozygous and heterozygous DD vs DI+II respectively. In the results of fixed effects method, D allele was associated with the onset of SRNS, but DD wasn't. However, in this study, significant heterogeneity among the included studies was observed for the analysis of D vs I or DD vs DI+II (*P* = 0.003, *P* = 0.0008; respectively; Table 4). The results coming from the random effects method might be more stable compared with those in the fixed effects method. In the analysis using the random effects method, we found that D allele and DD genotype were not associated with SRNS risk (D: OR = 1.60, *P* = 0.26; DD: OR = 1.90, *P* = 0.38; Figure 1-A for D, Figure 2-A for DD; Table 4). Furthermore, the fixed effects method was performed and we documented that there was significant association between II genotype and risk of SRNS relative to both other genotypes combined when the fixed effects method was used (OR = 0.51, *P* = 0.02; Table 4). Interestingly, II homozygous seemed not to play a protective role against the onset of SRNS when the random effects method was conducted (OR = 0.55, *P* = 0.23; Table 4). There was no significant heterogeneity for the comparison of II vs DD+DI, and the result from the fixed effects method might be more suitable for our investigation. The result for the comparison of II vs DD+DI was not stable and more investigations were needed to be performed to study this association.

**Table 4:** Meta analysis for the association of ACE I/D gene polymorphism with SRNS risk.

Comparisons	Studies number	heterogeneity P value	Fixed effects model		Random effects model	
			OR (95% CI)	*P*	OR (95% CI)	*P*
D vs I	5	0.003	1.38(1.04,1.83)	0.03	1.60(0.71,3.61)	0.26
DD vs (DI+II)	5	0.0008	1.26(0.84,1.90)	0.26	1.90(0.45,8.08)	0.38
II vs (DI+DD)	5	0.17	0.51(0.29,0.88)	0.02	0.55(0.20,1.47)	0.23
Sensitivity analysis						
D vs I	4	0.001	1.37(0.85,2.20)	0.20	2.11(0.51,8.66)	0.30
DD vs (DI+II)	4	0.0003	1.16(0.57,2.34)	0.68	2.46(0.20,29.84)	0.48
II vs (DI+DD)	4	0.08	0.47(0.19,1.15)	0.10	0.44(0.07,2.81)	0.39

In conclusion, as the result of that there were notable heterogeneities among the included studies for the comparisons of D vs I and DD vs (DI+II) (when significant statistical heterogeneity was observed among the included studies, the final results would come from those of the random effects models), the final results for the analysis of the association of ACE I/D gene polymorphism with SRNS susceptibility in Asian children were as follow: D: OR = 1.60, *P* = 0.26; DD: OR = 1.90, *P* = 0.38; II: OR = 0.51, *P* = 0.02 (Table 4).

#### Sensitivity analysis

The gene distribution of control group in the included study was not in HWE, which might be an important reason to cause heterogeneity to our investigation. In this meta-analysis, sensitivity analysis was performed. The genotype distributions of the control population in one study [8] didn't conform to HWE and this investigation was excluded from our study. Finally, four studies [4], [26], [27], [28] were recruited into our sensitivity analysis.

In the sensitivity analysis, the association of D allele or DD homozygous with SRNS susceptibility in Asian children wasn't observed when fixed effects method or random effects method was used (Table 4). Combination the results from sensitivity analysis with those in non-sensitivity analysis mentioned above, we might draw a stable conclusion that D allele and DD homozygous were not associated with SRNS susceptibility in Asian children.

The II genotype seemed not to play a protective role against SRNS risk in our sensitivity analysis when fixed effects method or random effects method was performed (Table 4). It was inconsistent with that in non-sensitivity analysis mentioned above, and the conclusion for II allele in our study was instable.

To sum up, as the result of that marked heterogeneity among the included studies were observed for all the comparisons (when a *P* value<0.10 for heterogeneity test among the included studies was observed, the final results would come from those in random effects models), the final results for the analysis of the association of ACE I/D gene polymorphism with SRNS susceptibility in Asian children were as follow: D: OR = 2.11, *P* = 0.30; DD: OR = 2.46, *P* = 0.48 ; II: OR = 0.44, *P* = 0.39 (Table 4).

#### 2.2 Association of ACE I/D gene polymorphism with SSNS susceptibility

When the fixed effects method was performed to explore the association between ACE I/D gene polymorphism and SSNS risk in Asian children, we found D allele and DD genotype were associated with the risk of SSNS when the fixed effects method was conducted. However, significant heterogeneity among the included studies was observed in the analysis for D allele or DD genotype (*P* = 0.002, *P* = 0.0002; respectively; Table 5). In the analysis using the random effects method, we found D allele and DD genotype were not associated with SSNS risk (D: OR = 1.24, *P* = 0.28; DD: OR = 1.72, *P* = 0.15; Figure 1-B for D, Figure 2-B for DD; Table 5). The results coming from the random effects method might be more stable compared with those in the fixed effects method.

**Table 5:** Meta analysis for the association of ACE I/D gene polymorphism with SSNS susceptibility.

Comparisons	Studies number	heterogeneity P value	Fixed effects model		Random effects model	
			OR (95% CI)	*P*	OR (95% CI)	*P*
D vs I	7	0.002	1.31(1.09,1.59)	0.005	1.24(0.84,1.84)	0.28
DD vs (DI+II)	7	0.0002	1.62(1.23,2.13)	0.0007	1.72(0.82,3.60)	0.15
II vs (DI+DD)	7	0.06	0.85(0.61,1.19)	0.35	0.95(0.55,1.63)	0.85
Sensitivity analysis						
D vs I	4	0.02	1.15(0.85,1.57)	0.36	1.34(0.76,2.38)	0.31
DD vs (DI+II)	4	0.0003	1.21(0.77,1.91)	0.40	2.04(0.52,7.97)	0.30
II vs (DI+DD)	4	0.18	0.83(0.48,1.44)	0.51	0.81(0.37,1.80)	0.61

Furthermore, the association of II genotype with SSNS risk in Asian children wasn't observed when the fixed effects method or the random effects method was used (*P* = 0.35, *P* = 0.85; respectively; Table 5). The result might be stable and II homozygous seemed not to play a protective role against the onset of SSNS in Asian children.

In conclusion, as the result of that remarkable heterogeneity among the included studies was observed for all the comparisons (when significant statistical heterogeneity was observed among the included studies, the final results would come from those of the random effects models), the final results for the analysis of the association of ACE I/D gene polymorphism with SSNS susceptibility in Asian children were as follow: D: OR = 1.24, *P* = 0.28; DD: OR = 1.72, *P* = 0.15; II: OR = 0.95, *P* = 0.85 (Table 5).

#### Sensitivity analysis

The gene distributions of control group in the included studies were not in HWE and those investigations were excluded from our sensitivity analysis. As a result, the gene distributions in control group of three studies [8], [14], [31] didn't conform to HWE and those studies were excluded from our study. Finally, four investigations [4], [26], [27], [28] were recruited into our sensitivity analysis.

In the sensitivity analysis, the association between D allele or DD homozygous and risk of SSNS in Asian children wasn't observed when fixed effects method or random effects method was conducted (Table 5). Combination the results coming from sensitivity analysis with those in non-sensitivity analysis mentioned above for SSNS, we might draw a stable conclusion that D allele and DD homozygous were not associated with SSNS susceptibility in Asian children.

The II genotype seemed not to play a protective role against SSNS onset in our sensitivity analysis when fixed effects method or random effects method was performed (Table 5). It was consistent with that in non-sensitivity analysis mentioned above for SSNS, and this conclusion for II allele was stable.

To sum up, as the result of that there was notable heterogeneity among the included studies for the comparisons of D vs I and DD vs (DI+II) (when a *P* value<0.10 for heterogeneity test among the included studies was observed, the final results would come from those in random effects models), the final results for the analysis of the association between ACE I/D gene polymorphism and SSNS risk in Asian children were as follow: D: OR = 1.34, *P* = 0.31; DD: OR = 2.04, *P* = 0.30; II: OR = 0.83, *P* = 0.51 (Table 5).

#### 2.3 The difference of ACE I/D gene distribution between SRNS and SSNS

**Figure 3:**
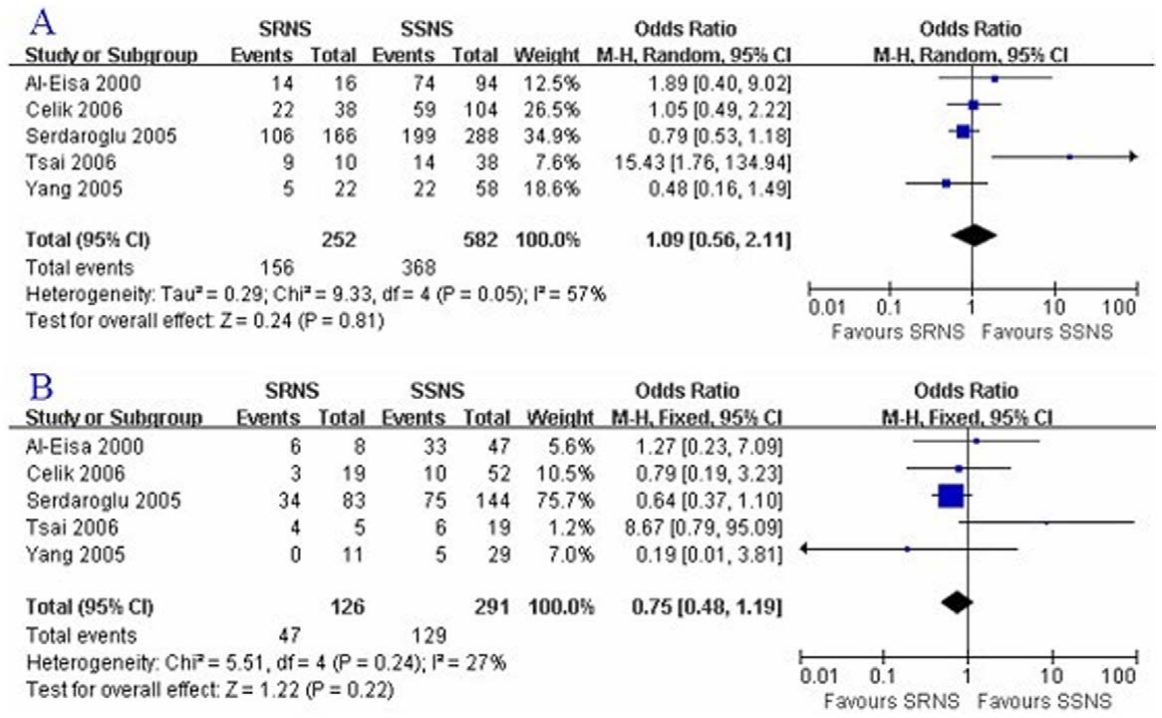
D allele or DD genotype distribution difference between SRNS and SSNS. A: D allele; B: DD genotype.

Five investigations [4], [8], [26], [27], [28] were recruited into this meta-analysis for the analysis of the difference of ACE I/D gene distribution between SRNS and SSNS in Asian children. The difference of ACE I/D gene distribution wasn't observed between SRNS and SSNS when fixed effects method or random effects method was used. The conclusion from the analysis of the difference of ACE I/D gene distribution between SRNS and SSNS was stable in this investigation. Consequently, as the result of that marked heterogeneity among the included studies was observed in the comparison of D vs I (when significant statistical heterogeneity was observed among the included studies, the final results would come from those of the random effects models), the pooled OR and *P* value for the difference in finally were as follow: D allele: OR = 1.09, *P* = 0.81; DD: OR = 0.75, *P* = 0.22; and II: OR = 0.77, *P* = 0.41(Figure 3-A for D, Figure 3-B for DD; Table 6).

**Table 6:** Meta analysis for the difference of the ACE I/D gene distribution between SRNS and SSNS.

Comparisons	Studies number	heterogeneity P value	Fixed effects model		Random effects model	
			OR (95% CI)	*P*	OR (95% CI)	*P*
D vs I	5	0.05	0.94(0.69,1.30)	0.72	1.09(0.56,2.11)	0.81
DD vs (DI+II)	5	0.24	0.75(0.48,1.19)	0.22	0.86(0.41,1.82)	0.69
II vs (DI+DD)	5	0.35	0.77(0.42,1.42)	0.41	0.83(0.39,1.76)	0.63

## Discussion

INS is the most common glomerular disease in children and represents a heterogeneous group of glomerular disorders. It can be divided into well-defined categories based on the response to standard prednisolone therapy. Early diagnosis for patient is very import for improving the prognosis in clinic. Up to now, there is no early diagnostic measure which can provide a reliable answer to predict the onset of SRNS. Renal histology detection is helpful to predict the clinical course of INS in childhood, but there are some limitations due to sampling, and the renal histology detection also can't accurately predict the response to corticosteroid treatment. Furthermore, it is also difficult to put in practice widely, especially in some developing country.

The genetic origin of renal diseases had been a focus of research in the past years; and some investigations found that the genetic alteration could become an early diagnosis indicator to predict the onset of some diseases [32], [33], [34]. There were some significant evidences showing that the RAS had taken part in the onset of some renal diseases [9], [10], [11], [12]. The level of plasma ACE, constitutively expressed in several types of somatic cells, is linked to an I/D polymorphism of 287 bp in intron 16 of the ACE gene [35], [36], [37]. D allele and DD homozygous have been reported to be associated with higher plasma ACE level [6], [16]. ACE is an important enzyme of RAS which can convert inactive angiotensin I into a vasoactive and aldosterone-stimulating peptide angiotensin II [38], [39]. The increased ACE protein expression is responsible for the elevation of plasma angiotensin II level [40]. So, D allele or DD homozygous might be an important molecular marker for early diagnosis of the onset of SRNS or SSNS. Most of the studies, investigating the association between ACE I/D gene polymorphism and the response to steroid treatment, were performed in Asian children with INS and tried to explore whether the ACE I/D gene polymorphism could become an early diagnosis indicator to predict the treatment response to steroid. However, data were insufficient. Furthermore, findings on the association of ACE I/D gene polymorphism with the susceptibility of SRNS or SSNS have been controversial since the first investigation was reported. In this study, we investigated whether the ACE I/D gene polymorphism could become a valuable indicator to predict the treatment response to steroid in Asian children with INS and tried to draw a more credible conclusion by meta-analysis.

In this investigation, we found the difference of association of D allele or DD genotype with the risk of SRNS or SSNS between case group and controls was not statistically significant. The results were in agreement with those of sensitive analysis. We also conducted the analysis for the difference of ACE I/D gene distribution between SRNS and SSNS, and found the difference of ACE I/D gene distributions between two groups were not statistically significant. Our results indicated that ACE D allele or DD genotype could not predict the treatment response to steroid in Asian children with INS. The conclusions were stable in our meta-analysis and they were consistent with the results of some included studies conducted in Asian children. Yang et al [28] reported the distributions of ACE I/D gene polymorphism were not significantly different between group of patient with INS and healthy control groups, and the distributions of ACE I/D gene polymorphism in the SSNS group were similar to those in the SRNS group, so we could draw a reasonable speculation that there was no significant association between ACE I/D gene polymorphism and the onset of SRNS or SSNS. Celik et al [27] observed that there was no significant association between ACE genotypes and risk of SSNS or SRNS, and they drew a conclusion that ACE I/D gene polymorphism did not contribute to the steroid response for patients with INS. Oktem et al [14] reported that there was no difference in the ACE I/D gene distribution between children with focal segmental glomerulosclerosis (most of them were SRNS) and normal controls. Serdaroglu et al [8] found that the distributions of ACE gene polymorphism in SSNS were similar to those of SRNS. Patil et al [29] reported that the frequency of DD genotype in SSNS patients was similar with that in controls.

However, some other Asian studies had an opinion that there was an association between ACE I/D gene polymorphism and treatment response to steroid. Al-Eisa et al [26] found that the INS cases with DD homozygous showed a significantly higher incidence for steroid sensitivity and steroid dependence. Tsai et al [4] reported that the higher incidence of DD genotype was observed in SSNS group and non-SSNS group, and a higher percentage of DD genotype in non-SSNS group and in SDNS group was also noted when compared with that in SSNS group. They drew a conclusion that DD homozygous might be a risk factor for INS and played an important role in the clinical response to steroids for INS patients. Dang et al [31] observed that the DD genotype distribution in non-SSNS patient was much higher when compared with that in SSNS and healthy control group and they drew the conclusion that ACE DD homozygous was associated with the treatment response of steroid in patient with INS.

Ethnic ingredient plays an important role in the epidemiology of INS [5]. In our investigation, we excluded those investigations conducted in Caucasians and Africans from this meta-analysis. Sasse et al [6] found there was no significant correlation between ACE genotype and steroid responsiveness in Swiss children with INS. Saber-Ayad et al [30] revealed that no association between ACE I/D gene polymorphism and risk of SSNS or non-SSNS by investigating the characteristics of ACE I/D gene distributions in Egyptian children with INS. The results of Sasse et al in Caucasian children and Saber-Ayad et al for African children were similar to those in our study, which was drew from Asian children with INS. However, in Africans, Fahmy et al [25] found that DD genotype might play a key role in the clinical response to steroid in Egyptian children with INS, but the difference of DD genotype between SSNS group and control group was not significant. For the analysis of DD homozygous, there was a significant difference between non-SSNS, SDNS and SRNS versus controls. For the analysis of D allele, a significant difference was observed between SSNS, SDNS and SRNS versus controls. The results of Fahmy et al [25] could draw a conclusion that ACE I/D gene polymorphism was associated with the treatment response of steroid in African children with INS. In conclusion, the role of ACE I/D gene polymorphism for SRNS and SSNS in Caucasian children and African children might be different from that in Asian children, and the number of the included studies was small and it was difficult to draw a stable conclusion for the association of ACE I/D gene polymorphism and the onset of SRNS or SSNS in Caucasian children and African children (only 1 report for Caucasian children and 2 investigations in African children). We excluded those investigations from our meta-analysis.

Our results indicated that there was no an association between ACE I/D gene polymorphism and the treatment response of steroid in Asian children with INS. The outcome might be stable. Our conclusion was inconsistent with those from the meta-analysis for the association between ACE I/D gene polymorphism and other diseases [20] [21] [23] [24] in Asian population. We speculated that the association of ACE I/D gene polymorphism with different diseases was different in Asians, and the ACE I/D gene polymorphism was not associated with the response to steroid treatment in Asian children with INS. However, those findings should be regarded cautiously because many other ingredients, such as heterogeneity of enrolled cases, limited statistical power, variable study designs and different interventions, were closely related to affect the results. Furthermore, whether the I/D gene polymorphism is just linked with other discrete loci involved in the occurrence of SRNS or SSNS is not clear at the moment.

In conclusion, the results in our study support that DD genotype or D allele is not associated with the susceptibility of SRNS or SSNS in Asian children, and DD genotype or D allele can't become a significant genetic molecular marker to predict the treatment response to steroid for children with INS. However, more case-control association investigations on larger, stratified populations are required to further clarify the role of the ACE I/D gene polymorphism in predicting the treatment response to glucocorticosteroid. More case-control investigations also should be conducted in Caucasians and African population for investigating the association between ACE I/D gene polymorphism and the response to steroid treatment in patient with INS.
